# Incidence and root causes of surgical site infections after gastrointestinal surgery at a public teaching hospital in Sudan

**DOI:** 10.1186/s13037-020-00272-4

**Published:** 2020-12-07

**Authors:** Rawan Sharaf Eldein Elamein Hassan, Sarah Osman Sayed Osman, Mohamed Abdulmonem Salih Aabdeen, Walid Elhaj Abdelrahim Mohamed, Razan Sharaf Eldein Elamein Hassan, Sagad Omer Obeid Mohamed

**Affiliations:** 1 Faculty of Medicine, University of Khartoum, Alqasr Avenue, P.O. Box 102, Khartoum, Sudan; 2 Faculty of Medicine, Nile Valley University, Khartoum, Sudan; 3 Faculty of Medicine, Alzaiem Alazhari University, Khartoum North, Sudan

**Keywords:** Surgical site infections, SSI, gastrointestinal tract, risk factors

## Abstract

**Background:**

Surgical site infections (SSIs) are common healthcare-associated infections and associated with prolonged hospital stays, additional financial burden, and significantly hamper the potential benefits of surgical interventions. Causes of SSIs are multi-factorials and patients undergoing gastrointestinal tract procedures carry a high risk of bacterial contamination. This study aimed to determine the prevalence, associated factors, and causing microorganisms of SSIs among patients undergoing gastrointestinal tract surgeries.

**Methods:**

A hospital based, cross-sectional study conducted at Soba University Hospital in Khartoum, Sudan. We included all patients from all age groups attending the gastrointestinal tract surgical unit between 1st September and 31st December 2017. We collected data about the socio-demographic characteristics, risk factors of SSI, and isolated microorganisms from patients with SSIs. A Chi-square test was conducted to determine the relationship between the independent categorical variables and the occurrence of SSI. The significance level for all analyses was set at *p* < .05.

**Results:**

A total of 80 participants were included in the study. The mean age was 51 +/- 16 years and most of the patients (67.5%) did not have any chronic illness prior to the surgical operation. Most of them (46.3%) of them underwent large bowel surgery. Twenty-two patients (27.5%) developed SSI post operatively and superficial SSI was the most common type of SSIs (81.8%). Occurrence of SSI was found to be associated with long operation time (*p* > .001), malignant nature of the disease (*p* > .001), intra-operative blood loss (*p* > .001), and intra-operative hypotension (*p* = .013). The most prevalent microorganism isolated from SSI patients was *E coli* (47.8%), followed by *Enterococcus fecalis* (13.0%) and combined *Pseudomonas aeruginosa + E coli* infection (13.0%).

**Conclusions:**

The results showed a high prevalence of SSIs among patients attending the gastrointestinal tract surgical unit and the most prevalent microorganism isolated from them was *E coli*. Measures should be taken to reduce the magnitude of SSI by mitigating the identified associated factors.

## Introduction

Surgical site infection (SSI) is the most frequent type of healthcare-associated infections, accounting for 14% − 25% of the total hospital-acquired infections [1, 2]. SSI is associated with a prolonged hospital stay, long-term disability, and additional financial burden, and significantly hampers the potential benefits of surgical interventions [3]. Notably, SSI is theoretically preventable but requires a particular investigation of early diagnosis and intervention [3].

The incidence of SSIs can vary across surgical procedures, specialties, and conditions, with a range of 0.1–50.4% as reported in a 2017-systematic review [4]. A prevalence survey in the UK National Health Service (NHS) indicated that approximately 8% of all patients (5743 out of 75,694 patients over a four-month period) admitted to hospital suffered healthcare-associated infections, with 15% of these infections being SSIs, and similar estimates have been found in France [3]. In Africa, the impact of SSIs ranged from 6.8–26% with predominance in the general surgeries [4].

Although there are global variations around the definition of an SSI, the European Commission classified SSIs into superficial incisional surgical site infection, deep incisional surgical site infection, or surgical site infection - organ/space, and the diagnostic criteria include the presence of one of the signs of infection (tenderness, swelling, reddening, and elevated skin temperature), purulent discharge from the incision site, and positive result of microbiological examination of material collected or after the surgical opening of the incision site [5, 6].

While the causes of SSIs are multi-factorial, recognized risk factors include the length of hospital stay, obesity, patient co-morbidities, duration and complexity of the surgery, and higher wound contamination classification [7]. The Centers for Disease Control and Prevention (CDC), classified wounds by their level of contamination as Clean (Class 1): which are non infective operative wounds in which no inflammation is encountered, with no involvement of respiratory, gastrointestinal, genitourinary tract, and oropharyngeal cavity; Clean-contaminated (Class 2): operative wounds in which either the respiratory, gastrointestinal or the genitourinary tract is entered under controlled conditions and with the only minor contamination, as resulted from operations involving the biliary tract, appendix, and oropharynx, provided no evidence of infection or a major break in sterile technique is encountered; Contaminated (Class 3): fresh, accidental wounds, resulting from operations with major breaks in sterile technique or gross spillage from the gastrointestinal tract, and incisions in which acute, non purulent (free from pus) inflammation is encountered; and Dirty (Class 4): old traumatic wounds with retained devitalized tissue and those that involve existing clinical infection or perforated viscera [8].

The risk of SSI is related to the level of contamination of the wound as demonstrated in a recent surveillance of surgical infections in NHS hospitals in England, which showed that the SSI risk following gastrointestinal tract procedures (especially large bowel surgery) reached 9.0% in 2018/19 [9]. While SSIs could be prevented by identifying and mitigating the predisposing factors, SSIs still have a significant burden on both the patient and health system in Sub Saharan Africa countries, where resources are limited, and the wound infection rates are higher than developed countries [10, 11]. Moreover, there is a knowledge gap regarding SSIs incidence, related factors and microorganism in Sudan. Therefore, this study aimed to determine the burden, associated factors, and the most prevalent microorganisms of SSIs among patients undergoing gastrointestinal tract surgeries.

## Materials and methods

### Study design and setting

A prospective, hospital based, descriptive cross-sectional study conducted at Soba University Hospital in Khartoum, Sudan during the period between 1st September and 31st December 2017. The Hospital is affiliated with the University of Khartoum and provides a host of therapeutic and diagnostic services at the highest level in Sudan through specialized units served by well trained healthcare staff. The current capacity of the hospital is approximately 500 beds and there are two general surgery units at the department of surgery. One of them is particularly specialized in the gastrointestinal and hepato-biliary conditions. Patients are booked for surgery from the referred clinics and then organized in the elective operations lists.

### Data collection and analysis

We included all patients from all age groups attending the gastrointestinal tract surgical unit during the study who underwent surgical procedures. Patients who died after admission to the hospital and before undergoing surgical procedures were excluded from the study. Each patient eligible for the inclusion in this study were enrolled consecutively and followed from the time of admission until time of discharge using a structured questionnaire, which was used to collect data adopted from a variety of literature.

The primary outcomes variable for this study were rate of SSI and type of the causing microorganisms. Diagnosis of SSI was made according to the after-mentioned criteria of the Centres for Diseases Control and Prevention. Data were collected from the medical records of patients including the operational and anesthetic sheets. We collected data about the socio-demographic characteristics, relevant clinical characteristics related to SSIs, and data about the isolated microorganisms from patients with SSIs.

Several patients’ clinical conditions have been described in the literature as risk factors to SSI [4, 12–28]. Based on the literature review and availability of the data, we assessed the following independent variables in this study: site of operation, type of operation, nature of the disease (benign vs. malignant), history of chronic illnesses (hypertension, heart diseases, and diabetes mellitus), surgical wound classification, duration of surgery, type of antibiotics prophylaxis, having bowel preparation prior to surgery, intra-operative blood loss, and intra-operative hypotension. After completion of data collection, data analysis was done using the SPSS software version 20 (SPSS Inc., Chicago, IL, USA). A Chi-square test was conducted to determine the relationship between the independent categorical variables and the occurrence of SSI. The significance level for all analyses was set at *p* < .05.

## Results

### General characteristics of the participants

A total of 80 participants were included in the study. Patients’ ages ranged from 20 to 98 years and the median age was 52 years (interquartile range: 25 years). 53.8% of them were males and 46.3% were females. Most of the patients (67.5%) did not have any chronic illness prior to the surgical operation and 46.3% of them underwent large bowel surgery. Thirty nine patients (48.8%) underwent clean-contaminated surgeries as well as another 39 patients underwent contaminated surgeries (Table 1).

Regarding preparation to surgery, all of patients received antibiotics prophylaxis preoperatively except one patient and near half of the patients (47.1%) received bowel preparation medications. Half of the patients (50.0%) received 2nd generation cephalosporin as a preoperative antibiotics prophylaxis, whilst 43.8% of them received both 2nd generation cephalosporin and Metronidazol as a preoperative antibiotics prophylaxis. However, most of them (81.2%) did not have documentation for the timing of administration of antibiotics prophylaxis preoperatively. Among the remaining who have complete data, (25%) and (62.5%) were given antibiotics 20 and 30 minutes before the operation, respectively. The majority (95.0%) did not receive second dose of antibiotics (Table 2).

### SSI prevalence, associated factors, and isolated microorganisms

Twenty-two patients (27.5%) developed SSI post operatively; eight patients developed SSI in the period of 3–4 days postoperatively, nine patients developed the infection in the period of 5–6 days postoperatively, and five developed the infection one week after the operation. Most of SSI cases (86.8%) were detected during hospital stay periods and the remaining cases were detected in the post-discharge follow-ups. Superficial SSI was the most common type of SSIs (82.6%).

Occurrence of SSI was found to be associated with long operation time of more than three hours (*p* > .001), malignant surgical diseases (*p* > .001), intra-operative blood loss (*p* > .001), and intra-operative hypotension (*p* = .013). The most prevalent microorganism isolated from SSI patients was *E. coli* (47.8%), followed by *Enterococcus fecalis* (13.0%) and combined *Pseudomonas aeruginosa* + *E. coli* infection (13.0%) (Table 3).

**Table 1:** Characteristics of the gastrointestinal surgical procedures done during the study period

Variables	Frequency	Percent
**Surgical condition**		
Non-malignant	52	65.0%
Malignant	28	35.0%
**Site of operation**		
Small bowel	16	20.0%
Large bowel	37	46.3%
Biliary	25	31.3%
Pancreatic	2	2.5%
**Indication for surgery**		
Elective	47	58.8%
Urgent	33	41.3%
**Underlying condition**		
Gallbladder / common bile duct stones	23	28.25%
Anal fistula	20	25%
Large bowel tumor	15	18.75%
Small bowel tumor	10	12.5%
Gastric tumor	6	7.5%
Gallbladder tumor	3	3.75%
Pancreatic tumor	3	3.75%
**Underlying condition**		
Laparoscopic cholecystectomy and stone extraction	23	28.25%
Examination under anesthesia and fistulectomy	20	25%
Bowel resection and anastomosis	25	31.25%
Total gastrectomy	6	7.5%
Radical cholecystectomy	3	3.75%
Whipple operation	3	3.75%
**Classification of surgery**		
Clean	2	2.5%
Clean-contaminated	39	48.8%
Contaminated	39	48.8%

**Table 2:** Risk factors associated with developing an SSI

Variables		Total	Patients with SSI	*P* value
**Sex**	Male	43 (50.4%)	13 (30.2%)	0.555
	Female	37 (49.7%)	9 (24.3%)	
**Medical comorbidities**	No	54 (67.5%)	12 (22.2%)	0.128
	Yes	26 (32.5%)	10 (38.5%)	
**Surgical condition**	Non malignant	52 (65.0%)	5 (9.60%)	> 0.001
	Malignant	28 (35.0%)	17 (60.7%)	
**Bowel preparation**	No	41 (51.3%)	9 (22.0%)	0.254
	Yes	39 (48.8%)	13 (33.3%)	
**Second antibiotic dose**	No	76 (95.0%)	20 (26.3%)	0.301
	Yes	4 (5.0%)	2 (50.0%)	
**Site of operation**	Small bowel	16 (20.0%)	8 (50.0%)	0.074
	Large bowel	37 (46.3%)	6 (16.2%)	
	Biliary	25 (31.3%)	7 (28.0%)	
	Pancreatic	2 (2.5%)	1 (50.0%)	
**Classification of surgery**	Clean	2 (2.5%)	1 (50.0%)	0.746
	Clean-contaminated	39 (48.8%)	11 (28.2%)	
	Contaminated	39 (48.8%)	10 (25.6%)	
**Intra-operative hypotension**	No	39 (48.8%)	6 (15.4%)	0.018
	Yes	41 (51.3%)	16 (39.0%)	
**Duration of operation**	Less than 3 hours	60 (70.6%)	8 (14.3%)	> 0.001
	More than 3 hours	25 (29.4%)	14 (58.3%)	
**Intra-operative blood loss**	No blood loss	48 (60.0%)	4 (8.30%)	> 0.001
	Less than 500 ml	18 (22.5%)	9 (50.0%)	
	More than 500 ml	7 (8.5%)	3 (42.9%)	
**Type of antibiotics prophylaxis**	2nd generation cephalosporin	40 (50.0%)	10 (25.0%)	0.383
	Metronidazol + 2nd generation cephalosporin	35 (43.8%)	11 (31.4%)	
	Metronidazol	4 (5%)	0

**Table 3:** Features and types of microorganisms isolated from patients with SSI

Variables		Total
**Type of SSI**	Superficial	19 (82.6%)
	Deep	3 (13%)
	Organ/space	1 (4.3%)
**Timing of SSI relative to procedure**	Day 3–4	8 (34.8%)
	Day 5–6	9 (39.1%)
	One week and more	6 (26.1%)
**When was SSI detected**	During admission	19 (86.4%)
	After discharge	3 (13.6%)
**Bacteria**	*E coli*	11 (47.8%)
	*Enterococcous fecalis*	3 (13.0%)
	*Pseudomonas + E coli*	3 (13.0%)
	*Enterococcous + Klebsiella*	1 (4.30%)
	*E coli + Klebsiella*	2 (8.70%)
	*Staph. aureus + Pseudomonas*	1 (4.30%)
	*E coli + Enterococcous fecalis*	1 (4.30%)

## Discussion

Reducing the risk of SSIs is dependent on identifying the risk factors as well as developing the needed measures for prevention and management [29]. An artificial neural network analysis for prediction of SSIs showed that the interaction between the risk factors is complex and depicted that some factors, such as preoperative white blood cell count and wound classification, could be more important than other variables [30].

The results of this study showed that 27.1% of the patients had SSI. Several risk factors were found to be significantly associated with the development of SSI including: malignant nature of the disease, intra-operative blood loss, intra-operative hypotension, and long operation time. Majority of the patients had superficial wound infections, which were discovered mostly during post-operative hospital stay with drainage from the wound site 5–6 days post operatively. Those patients with SSIs were handled according to the standard guidelines and wound dressing twice per day was offered for patients with SSI.

The higher risk of acquiring SSI among patients with malignant diseases compared to patients with benign diseases is reasonable since malignancy is associated with weak immune system and vulnerability to various infections, and it is consistent with studies reported that SSI was observed in 30–60% of patients after colorectal cancer surgeries [5, 16, 17]. The lack of association between SSI and timing of antibiotics prophylaxis in this study is similar to another retrograde cohort study [31]. According to the standard guidelines, antibiotics prophylaxis should be given 60 minutes before the incision in most of types of antibiotics and doubling of the dose should be considered when the duration of operation exceeds 4 hours [32].

There was a significant association between SSI and intra-operative blood loss of more than 500 ml like another study showed the 26.1% of those who had massive intra-operative blood loss developed wound infection [18]. This is relevant since blood loss is directly related to decreased tissue oxygenation and aiding in the development of SSI. Also, intra-operative hypotension is found to be strongly related to SSI as previously reported [19, 20], and this is related to the poor wound perfusion resulting from intra-operative hypotension.

The association between SSI and long duration of operation of more than 180 min match a systematic review that showed the same results in most of its studies [21]. It has been demonstrated that there was an 80% increase in likelihood of SSI with surgeries longer (versus shorter) than three hours [4]. Prolonged operative time allows time for over- handling the wound edges and also contact with contaminated fluids coming out of the surgical field.

The most prevalent micro-organisms isolated were *E coli* and *Enterococcus fecalis*, whilst other studies showed that *Staphylococcus aureus* was the most common isolated bacteria from different wound types [33, 34]. A recent meta-analysis found that 30.6% of SSI cases in Ethiopia were caused by *Staphylococcus aureus*, which was like other reports from India, Nigeria, and Uganda [35]. Also, *Pseudomonas aeruginosa* are commonly isolated in infected wounds following surgeries and burns, whereas *Enterobacteriaceae* are commonly isolated from wounds in immune compromised patients and abdominal surgeries [33, 34]. These virulent isolated microorganisms need to be put in high consideration and further elaboration to be done regarding this issue.

## Conclusion

The results showed a high prevalence of SSI among patients attending the gastrointestinal tract surgical unit and it was associated with the malignant diseases, intra-operative blood loss, intra-operative hypotension, presence of surgical drains, and long operative duration. The most prevalent microorganisms isolated were *E coli, Enterococcus fecalis* and *Pseudomonas aeruginosa*. Measures should be taken to decrease intra-operative blood loss, reduce the waste of time due to any cause intra-operatively so as to reduce the overall duration of operation.

## Data Availability

The datasets used during the current study are available from the corresponding author on reasonable request.

## Abbreviation

SSI: Surgical site infections
